# What You Gain and What You Lose in COVID-19: Perception of Medical Students on their Education

**DOI:** 10.6061/clinics/2020/e2133

**Published:** 2020-07-06

**Authors:** Lucas Albuquerque Chinelatto, Thamara Rodrigues da Costa, Vitor Macedo Brito Medeiros, Gustavo Henrique Pereira Boog, Flávio Carneiro Hojaij, Patricia Zen Tempski, Milton de Arruda Martins

**Affiliations:** Faculdade de Medicina FMUSP, Universidade de Sao Paulo, Sao Paulo, SP, BR

## CONTEXT

The confirmation of a pandemic by the World Health Organization on March 11, 2020 proved that the SARS-CoV-2 virus would become a worldwide health issue (1). The Brazilian situation was no different from other countries: there was pressure on the health system and health workers. According to an article published by former Brazilian Health Ministry members, the country has been preparing itself for the COVID-19 disease since January 10, 2020 (2), and in many cities and states, isolation protocols have been set up (3). Even so, the number of cases and deaths keep rising in the country, counting 514,849 official cases and 29,314 deaths on June 1, 2020 (4).

Many adaptations are being made in the way society works. Medical training had to be adapted in this context. In many schools, the internship was interrupted while other educational activities were adapted to virtual learning environments, with the introduction of web-based activities and new teaching technologies (5,6). These resources, despite being already present in many schools, became the only way of teaching due to isolation protocols demanding many adaptations (7).

In Brazil, on the same hand as what was proposed by the Association of American Medical Colleges (8), 1^st^ to the 4^th^ years of medical school had their on-site activities suspended. The 5^th^ and 6^th^ years, our internship students, are still going to their university-hospitals, as interns work as health-staff in many situations. Their engagement was endorsed by the Federal Government COVID-19 combat strategy (9), which suggests using the internship labor force for the epidemic combat, in similar ways that are proposed by others (10,11). At the same time, a provisory measurement taken by the president allowed medical students to graduate with 75% completion from their internship (12). In the Faculty of Medicine of the University of São Paulo, graduation has been divided in four main educational strategies.

## FMUSP EDUCATIONAL CRISIS STRATEGIES

First to third years’ organization was similar, as they are based mainly on theory expository classes. Although there are some practical lessons, they do not reach more than 25% on those year’s schedules. Teaching activities had the support from the Medical Education Development Center (CEDEM), in order to review courses' educational outcomes, adapt educational strategies and bring classes to online platforms. A two-week break occurred to allow teachers to organize their courses to the new educational strategies and resources. Activities were divided in two main ways: synchronous and asynchronous. The first are live transmissions that allow teacher-student interaction. The other ones are on-line texts, articles, or recorded classes. For questions, many teachers have an online forum to keep track of students’ doubts.

The 4^th^ year, also known as ‘pre-internship’, which is essentially a 50% theory and 50% practice year, became, despite its characteristics, a completely online course. Learning dynamics in this year are very specific, as undergraduates are divided into 8 groups and they rotate in different medical areas. In order to respect any difficulties that the undergraduates could have in this crisis time, some student assessments were suspended and others were restructured into Internet based works. Practical activities, on the other hand, have been cancelled and will be resumed later.

The most disruptive situation happened in the 5^th^ year of medical school. Many activities of the interns are in ambulatories or elective surgeries, which were cancelled in our university hospital complex (HCFMUSP), as it became a center for the care of COVID-19 patients. Given this situation, students and the CEDEM - with the participation of the president of the graduation committee - discussed alternatives. They agreed that, for this specific year, the best would be to cancel activities, as their quality would be too jeopardized if they were continued as usual, or flipped to online activities. At the same time, a volunteering project was organized by CEDEM, which allowed students to work in medical education organization activities, as well as research on COVID-19 and health care assistance. The volunteering project was based on medical education recommendations (6,10,11), which suggested that students could engage in COVID-19 care. It was proposed for them to act in areas related to their academic competences, while it was guaranteed that they would have medical supervision, adequate personal protective equipment and allowance to leave their project at any time. The volunteering program was then also extended for 4^th^ and 6^th^ year students.

The last year of medical school, on the other hand, had their activities maintained, as interns act on emergency sectors from HCFMUSP. Most of the clerkships have not changed at all, while others had some minor adaptations. In order to minimize the impact on learning quality, there is a meeting with students’ representatives and the undergraduate committee every 2 weeks to discuss the current situation.

## WHAT WE HAVE LOST

Virtual lectures quality is apparently much correlated to previous medical area knowledge on internet-based teaching resources. Some of them already used recorded videos and therefore it was easier to make a transition to online classes. Some teachers are still having trouble with the new technologies, such as planning for videoconferences, or using interactive methods through web services. Even in well-adapted courses, structuring clinical case discussions, a pillar on medical teaching, to a virtual classroom proved to be very hard, demanding great effort in interacting from both teacher and students. This, unfortunately, adds up to a decrease in class discussions quantity and quality.

Many students are anxious about when they will write their tests and how precisely they will be graded, as there is no clear projection about when classes will go back to usual - since there may be months of social isolation followed by a long period of time until the health care system is once again reorganized. Anxiety also rises in relation to how the medical school will be able to make up for the lost practical classes. Those lessons are essential to medical learning and many students worry about not being adequately prepared to act as interns in the future.

For students training at the university hospital (last year of medical school), there are, unfortunately, some downsides too. The number of emergencies decreased overall and many of them became COVID-19 related. This transformed into insecurity and anxiety among students, as they will become doctors in less than eight months, and they are not sure if they will be prepared for other kinds of emergencies.

The main feelings from undergraduates are anxiety and powerlessness, in the face of the current health crisis. Most negative aspects are some problems which were already present in medical training (heterogeneity in class quality, fear of not becoming a good doctor), but were amplified by the current situation. What might be the greatest loss is not directly related to teaching and learning. Extensions, parties, group projects and even lunch or a break in the cafeteria are part of the college period of life, and, unfortunately cannot be lived right now. One cannot disregard the importance of community life during graduation for good academic formation. Of course, the whole society is suffering from social isolation, but the impacts of the lack of human contact in a medical school and its consequences cannot be ignored.

## WHAT WE HAVE WON

Overall, the current situation resulted in more free time for students. There is less commuting time, a significant reduction in scheduled classes, and a great deal of extracurricular activities were cancelled. With this free time, some students are studying for the medical residence selection test. Others are investing in their mental or physical health. Some are even developing new hobbies. For instance, during mentoring encounters (the faculty has a mentor program, with one graduated mentor for a group of 10-20 students) many undergraduates reported new habits during the quarantine, such as: reading, cooking, playing instruments, painting, reading novels, meditating and practicing physical activity at home.

At the same time, a great engagement with the crisis management can be seen: 311 students volunteered to help with the current crisis. The volunteer program gave an opportunity for many students, that already had sufficient theory knowledge, to get more involved in medical education, research, and health care without the need to worry about tests or doing everything quickly, as they are used to in their regular working environment.

Asynchronous classes also end up having positive aspects. They allow students to organize their own study time, and therefore develop time management skills. The last year even reports an increase of quality in some of their clinical stages, as there are now fewer people working in the hospital, therefore, they can do more procedures.

One must also recognize the efforts being made by our medical school to offer a good quality undergraduate program, even in such adverse situation. The university, for example, distributed internet SIM-cards for those students that did not have internet access in their homes. Many teachers also improved their knowledge, with the aid of CEDEM and volunteer students, on how to use online platforms and on how to match their classes to their learning objectives.

## CONCLUSION

All students understand that this is an exceptional circumstance, and therefore there will be some educational losses. Despite the negative impacts of this medical education turmoil, exceptional times also represent an opportunity for changes. Online education can be helpful and introducing it as part of the curriculum may allow more free time for students, and may teach them time management skills. Despite that, the virtual environment also allows new opportunities for teaching and learning. At the same time, the volunteering experiences bring attention to the value of non-graded elective courses to make student’s knowledge more diverse and increase their motivation in learning without worrying about grades.

The 21^st^ century will be divided into before, and after, the COVID-19 era. It relies on us, as a community, to make the best out of this pandemic and transform the post-COVID-19 era into a better one.

## AUTHOR CONTRIBUTIONS

Chinelatto LA wrote the manuscript drafting and final version. Costa TR, Medeiros VMB and Boog GHP assisted in the manuscript drafting and writing. Hojaij FC and Tempski PZ supervisioned the manuscript drafting and writing and assisted in the manuscript final version. Martins MA reviewed the manuscript final version. All of the authors contributed to the manuscript content.
